# A New Way of Taking Cod Liver Oil

**Published:** 1852-10

**Authors:** 


*A New Way of Taking Cod-liver Oil.*

Dr. Beneditti recommends the following means for disguising the nauseous taste of cod-liver oil:—Make a paste with the oil and powdered starch of arrow-root, and prepare the bolus by wrapping it in a moistened wafer. About sixteen of such boluses night and morning suffice in the beginning; more may subsequently be taken, or they may be made larger, as the swallowing of them becomes easy by habit.—*Ibid.*

